# Cascade Dielectrophoretic Separation for Selective Enrichment of Polyhydroxybutyrate (PHB)-Producing Cyanobacterium *Synechocystis* sp. PCC 6803

**DOI:** 10.3390/mi16121402

**Published:** 2025-12-12

**Authors:** Songyuan Yan, Sara Louise Pacheco, Asa K. Laskie, Cesar Raul Gonzalez Esquer, Lawrence Kulinsky

**Affiliations:** 1 Samueli School of Engineering, University of California, Irvine, CA 92617, USA; 2 Los Alamos National Laboratory, Bioscience Division, Microbial and Biome Sciences Group, Los Alamos, NM 87545, USA; 3 Interdisciplinary Life Sciences Graduate Programs, University of Texas at Austin, Austin, TX 78712, USA

**Keywords:** electrokinetics, microfluidics, biomanufacturing

## Abstract

Maintaining favorable biological productivities in photosynthetic biomanufacturing systems, especially when the risk of contamination with competing microbes is high, remains a challenge to achieve while maintaining economic feasibility. This study presents a dielectrophoresis (DEP)-based microfluidic approach for isolating a desired strain within a co-culture. The cyanobacterium *Synechocystis* sp. PCC 6803 (a strain capable of producing the bioplastic precursor polyhydroxybutyrate, or PHB) was enriched from mixed cultures containing the competing cyanobacterium Synechococcus elongatus PCC 7942 (which does not naturally produce PHB). A DEP cascade electrode system was established to increase purification efficiency through sequential enrichment, which leveraged inherent differences in cell morphology and dielectric properties, to achieve the selective separation of these strains under physiological conditions. A substantial increase in the relative abundance of PHB-producing cells was assessed by optical microscopy and flow cytometry characterization, confirming more than five-fold reduction of the Synechococcus fraction in the refined cell mix. The presented electrokinetic platform offers a scalable and effective approach for selectively enhancing desired microbial components within microbial biomanufacturing systems, leading towards improved product yields.

## 1. Introduction

Microbial systems have long been utilized in biomanufacturing applications as catalysts for the synthesis of various commodities, including food, fuel, therapeutics, enzymes, and chemicals [1,2,3,4,5]. While monoculture has traditionally been applied to industrial processes, there is an increasing interest in developing and controlling microbial communities for improved process efficiency [6,7]. However, the complex dynamics within a microbial community represent a persistent challenge that can significantly influence overall process efficiency and product yield [6,8,9]. For example, selective pressures during intensified cultivation conditions may adversely affect the retention of high-performing strains in the microbial mixture [6,10]. Therefore, the development of scalable, selective, and high-throughput cell separation strategies is essential for improving the performance and reliability of microbial biomanufacturing systems.

**Figure 1:**
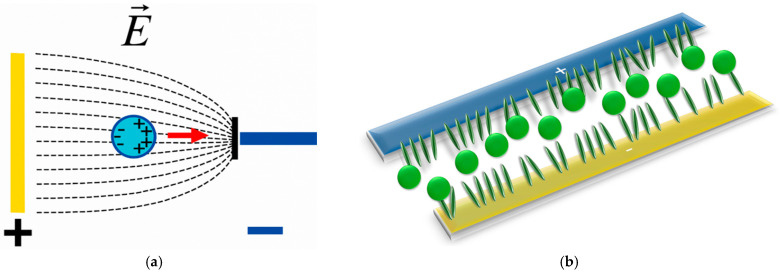
Dielectrophoresis (DEP) for the separation of cells within a population. (**a**) Schematic representing polarization-induced particle movement within a non-uniform electric field. (**b**) Representation of electrokinetic separation between two different cyanobacterial species—elongated *Synechococcus* cells and spherical *Synechocystis* cells where the elongated cells are attracted to the electrodes, while the spherical cells are repelled from the electrodes.

Existing cell separation methods, such as fluorescence-activated cell sorting (FACS), offer high specificity but suffer from limited scalability, limiting their use in industrial bioprocessing. Scalable alternatives, such as centrifugation, filtration, and the use of selective growth media or antibiotics, are commonly employed in biomanufacturing but often lack the specificity required to isolate closely related cell types or subpopulations [11,12,13]. In contrast, electrokinetic separation offers a scalable solution to cell purification challenges. This technique can distinguish cell types in continuous-flow and high-throughput systems, making it suitable for industrial applications [9,14,15,16,17,18]. Among electrokinetic methods, dielectrophoresis (DEP) is particularly effective due to its dependence on dielectric contrast between cells and the suspending medium [17]. DEP functions by applying a non-uniform electric field to polarizable cells (Figure 1a). When exposed to such a field, particles or cells are induced to develop dipole moments, thereby generating a net force. The direction and magnitude of this force depend on the relative permittivity and conductivity of the particles compared to the medium. Particles with higher effective polarizability than the medium experience positive DEP (pDEP) and are attracted toward regions of higher electric field gradients. In contrast, in negative dielectrophoresis (nDEP), particles are repelled from regions of higher electric field gradient (Figure 1b). In alternating current DEP (AC-DEP), the force’s direction and magnitude can be tuned by adjusting the frequency of the applied AC field, allowing for frequency-dependent discrimination of target cells [17]. This enables selective enrichment of cell populations with distinct shapes or electrical properties, potentially contributing to improved process efficiency and product yield in microbial biofuel production systems.

The utilization of DEP is demonstrated in this study. An artificially designed photosynthetic biomanufacturing consortium is comprised of two cyanobacterial strains: *Synechocystis* sp. PCC 6803 (hereafter *Synechocystis*) and *Synechococcus elongatus* PCC 7942 (hereafter *Synechococcus*). While both strains have been previously utilized as potential biomanufacturing platforms for the production of renewable chemicals, they have distinct morphological and physiological properties [19]. *Synechocystis* has a spherical morphology and a natural capacity to produce the bioplastic precursor polyhydroxybutyrate (PHB), while *Synechoccocus* is elongated and lacks the ability to produce precursor PHB [20,21,22]. Therefore, this strain pair provides a well-controlled system to test the specificity of DEP-based separation, particularly the ability to enrich productive cells (in this case, PHB) while removing non-productive ones. Here, we identified an AC frequency range where the *Synechocystis* and *Synechoccocus* strains experience opposing DEP forces, allowing for their efficient separation within their standard growth media. This evidence suggests that DEP is a suitable method for manipulating biomass productivity in a photosynthetic mixed culture, which is essential for effective biomanufacturing.

## 2. Materials and Methods

### 2.1. Physics and Simulation of the Dielectrophoretic Separation

The governing equation of the DEP force experienced by a polarizable spherical particle in a non-uniform electric field is represented by Equations (1)–(3) [23,24]:

(1) $$F_{\mathrm{DEP}}=2\pi r^{3}\varepsilon_{m}Re\left\{CM\left(\omega \right)\right\}\nabla {\left|E\right|}^{2}$$

(2) $$CM\left(\omega \right)=\left\{\frac{\varepsilon_{p}^{*}\;\left(\omega \right)-\varepsilon_{m\;}^{*}\left(\omega \right)}{\varepsilon_{p}^{*}\;\left(\omega \right)+2\varepsilon_{m}^{*}\left(\omega \right)}\right\}$$

(3) $$\varepsilon^{*}\left(\omega \right)=\varepsilon -i\frac{\sigma }{\omega }$$

where r is the radius of the particle, ε_m_ is the relative permittivity of the medium, Re{CM(ω)} is the real part of the Clausius–Mossotti (CM) Factor, ∇|E|^2^ is the gradient of the magnitude of the electric field squared, ε_m_* and ε_p_* are the complex permittivities of the fluidic medium and the particle as stated in Equation (3), *i* is the imaginary number, *i* = $\sqrt{-1}$, and σ is the electrical conductivity. The subscripts *p* and *m* in Equation (2) identify particles and media, respectively. The polarizability of the particle and media is dependent on the CM factor, which is a function of the supplied AC frequency ω. To simulate the DEP behavior of the elongated *Synechococcus*, the particles were modeled as prolate ellipses. The time-averaged DEP force in two dimensions for such an anisotropic particle is expressed as [23,24,25]:

(4) $$F_{DEP}=\frac{1}{2}\pi \varepsilon_{0}\left[ab^{2}Re\left(K_{∥}\right)\nabla \left(E_{∥}^{2}\right)+ba^{2}Re(K_{⊥})\nabla (E_{⊥}^{2})\right]$$

In this equation, *ε*_0_ is the permittivity of free space. The terms *a* and *b* define the geometry of the elliptical cell, where *a* corresponds to the semi-major axis (along with the cell’s length) and *b* to the semi-minor axis (across the width). The electric field components $E_{∥}$ and $E_{⊥}$ represent the field strength along and perpendicular to the particle’s long axis, respectively. The gradients ∇($E_{∥}^{2}$) and ∇$E_{⊥}^{2}$ describe how these squared field components change in space, contributing to the magnitude and direction of the resulting DEP force. The terms $K_{∥}$ and $K_{⊥}$ represent the complex CM factors along the respective directions, accounting for the relative polarizability of the particle compared to the surrounding medium. Taking the real part of these CM factors determines whether the particle experiences pDEP or nDEP.

A two-dimensional finite-element model was developed in COMSOL Multiphysics 6.2 (COMSOL, Inc., Burlington, MA, USA) to simulate the DEP behavior of elongated *Synechococcus* in an AC electric field. The simulation domain was constructed using four subdomains: three ellipses representing elongated particles and one square fluid domain (30 μm × 30 μm) that encompasses the electrodes. Each elliptical particle had a semi-major axis of 3 μm and a semi-minor axis of 0.5 μm. The ellipses were placed at different *x*-axis positions (5 μm, 15 μm, and 25 μm) and oriented at rotation angles of 0°, 60°, and 90°, respectively, to simulate various alignment conditions with respect to the electric field. Similarly, to simulate the DEP behavior of spherical *Synechocystis* in an AC electric field, a 3 μm diameter circle was used to model the spherical strain of cells in the identical electric field, while adopting the DEP force from Equations (1)–(3).

The materials assigned to the domains represented the distinct dielectric and conductive properties of the particles and the medium. The aqueous medium was assigned relative permittivity of 80 and a conductivity of 0.01 S/m (typical values for water-based solutions). The particles were assigned a permittivity of 50 and a higher conductivity of 0.5 S/m [26,27,28]. These material properties were incorporated through the AC/DC Module using frequency-domain dielectric models.

The governing physics was defined using the Electric Currents interface. A sinusoidal potential of 10 V at 10 MHz was applied to one electrode, while the opposing electrode was grounded. All other boundaries were electrically insulated. The model also included a Creeping Flow module with stationary, no-slip boundary conditions and a zero initial velocity to simulate near-stationary fluid. The 30 µm × 30 µm domain was selected to represent the vicinity of the electrode edges, where the DEP force is the strongest and most relevant to cell–electrode interactions. This domain size preserves the local electric-field environment experienced by the cells.

The Clausius–Mossotti factors and crossover-frequency characteristics of *Synechocystis* and *Synechococcus* in BG-11 are not available in the literature, limiting the ability to identify the separation frequency through CM-based prediction. A broad frequency sweep was therefore conducted, which showed that 10 MHz at 20 Vpp produced the most consistent contrast in DEP response, yielding strong pDEP attraction of *Synechococcus* and a weak nDEP response of *Synechocystis* while maintaining cell viability. These empirically validated operating conditions provided reproducible separation across replicates and were used throughout the study.

### 2.2. Strain and Cultivation Conditions

*Synechococcus elongatus* PCC 7942 was maintained in solid BG-11 media supplemented with 10 mM TES pH 8.0 and *Synechocystis* sp. PCC 6803 in solid BG-11 media [29] supplemented with TES pH 8.0 and 5 mM glucose, both kept at 25 °C and 10 μmoles photons m^−2^ s^−1^. For cultivation, cells were grown in 125 mL Erlenmeyer flasks containing 40 mL BG-11 (Thermo Fisher Scientific, Waltham, MA, USA) supplemented with 10 mM TES pH 8 and 10mM NaHCO_3_, shaken at 130 rpm and grown under continuous light (60 μmoles photons m^−2^ s^−1^) and 30 °C. Growth was tracked via optical density measurements at 730 nm. Samples were harvested when OD_730_ reached between 0.5–0.8 and stored at 4 °C until used. Experimental mix of *Synechocystis/Synechococcus* was prepared via manual mixing of 2:1 *Synechocystis* to *Synechococcus* by volume. The mix was characterized by using cell counts obtained by flow cytometry (see below).

For viability assay, post separation samples (~10 μL) were pipetted on to a BG-11 plate supplemented with 10mM TES pH 8 and 5mM glucose. Cells were spread around the plate with a 10ul inoculating loop and placed under 150 μmoles photons m^−2^ s^−1^ under ambient air and 25 °C.

### 2.3. Electrode Fabrication

**Figure 2:**
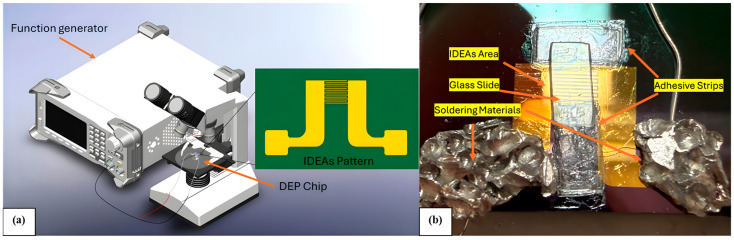
DEP experimental setup. (**a**) Schematic showing the function generator connected to a microscope-mounted chip; the inset includes the IDEAs pattern used for cell separation. (**b**) Close-up of the chip covered by a glass slide, with labeled IDEAs area, soldered connections, and adhesive mounting.

Gold interdigitated electrode arrays (IDEAs) were microfabricated on 4-inch silicon wafers using standard lithography. The electrode pattern is illustrated in Figure 2a,b. The gaps between the interdigitated electrodes are 80 μm in width. The microfabrication sequence included a Shipley 1827 photoresist (Shipley Company, LLC, Marlborough, MA, USA) layer spin-coated onto the surface at 3000 rpm for 30 s using the Laurell photoresist spinner followed by a soft-bake at 95 °C for 30 min. The resist was exposed through a patterned photomask (CadArt, Inc., Bandon, OR, USA) by 10 mW/cm^2^ dose of UV light for 35 s using a Karl Suss MA6 Mid/Deep UV Mask Aligner (SUSS MicroTec, Garching bei München, Germany). The resist was developed in MF-319 developer (Shipley Company LLC., MA, USA). Next, a 500 Å Chromium (Cr) adhesion layer from 99.95% chromium granules (Kurt J. Lesker Company, Jefferson Hills, PA, USA) was deposited onto the wafer using a CHA-600S/CV-8 thermal e-beam evaporator (Temescal Systems, Livermore, CA, USA), followed by a deposition of a 2500 Å thick Gold (Au) layer from 99.99% gold pellets (Kurt J. Lesker Company, Jefferson Hills, PA, USA) using the e-beam evaporation. The sample underwent a lift-off process using an acetone solution submerged in an ultrasonic bath for 10 s with a Branson CPX2800H Ultrasonic Digital Bench (Emerson Electric Co., St. Louis, MO, USA) to create a set of interdigitated gold IDEAs.

The experimental setup is illustrated on Figure 2. The IDEAs were connected to bus wires (All Electronics, Van Nuys, CA, USA) using indium solder at the contact pads on both ends of the electrodes. An enclosure for the suspension of the cells placed on top of the IDEAs was cut out of a double-sided adhesive tape (FLEXcon Company, Inc., Spencer, MA, USA) and 4 layers of the 75 μm-thick double-sided tape strips were affixed around the interdigitated region. A 3 μL droplet of the prepared cell suspension in BG-11 growth medium was then pipetted into the central window of the device. A glass coverslip (Fisherbrand, Thermo Fisher Scientific, Waltham, MA, USA) was placed over the droplet to minimize evaporation and enhance imaging quality. The buss wires connected the IDEAs to a function generator (Stanford Research Systems, Sunnyvale, CA, USA), configured to deliver the desired AC frequency, with a 20 V peak-to-peak voltage. Cell motion was observed using a Nikon Eclipse microscope (Nikon, Tokyo, Japan), and the image acquisition was carried out using a SPOT RT sCMOS Camera (Diagnostic Instruments, Inc., Sterling Heights, MI, USA). Video recordings were made using SPOT Basic software (SPOT Imaging, Sterling Heights, MI, USA) and supplemented with screen captures using Camtasia Recorder (TechSmith, Okemos, MI, USA).

### 2.4. Electrokinetic Separation Cascade

**Figure 3:**
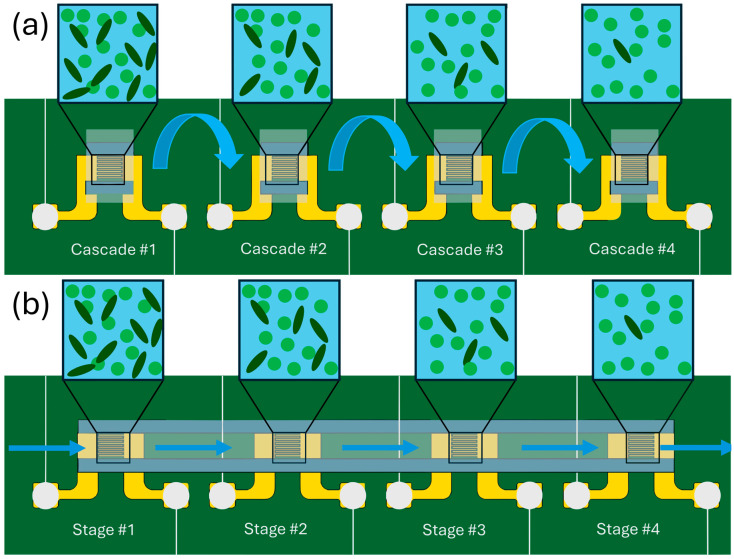
Schematic representation of a multi-stage cascade for selective cell separation using (**a**) serial cascade modules, and (**b**) quasi-continuous flow (QCF) modules. In serial cascade the separation takes place after 3-min dwell time of cell suspension on the electrodes before the cell suspension is pipetted out and transferred to the next stage. In the QCF approach that emulates the flow passing through sequential stages integrated in the same flow path, the dwell time is reduced to five seconds.

To achieve stepwise purification of *Synechocystis* from a mixed *Synechocystis*/*Synechococcus* solution, a cascade consisting of sequential IDEAs was integrated into a microfluidic platform, as illustrated in Figure 3. Four individual chips each containing an interdigitated electrode array were placed next to each other. In the serial separation experiments, 3 μL of the prepared solution was pipetted into the interdigitated electrode area and allowed to remain for 3 min to enable sufficient dielectrophoretic interaction. The solution was pipetted out after 3 min and transferred to the next electrode. After each aspiration step 2.5 μL (rather than 3 μL) were pipetted out due to evaporation and dead volume and the media was added to sum up to 3 μL of total volume. The process was repeated for the selected number of times as indicated in the Results and Discussion below. This facilitated progressive separation and enrichment of *Synechocystis*. In contrast, for the quasi-continuous flow (QCF) approach, the dwell time of the cell suspension on each IDEAs chip was reduced to five second before the cell suspension was pipetted to the next chip. QCF approach emulates the continuous flow in the microfluidic device where IDEAs are placed one after another. This configuration allowed simultaneous processing of larger sample volumes and enabled comparison of separation performance under dynamic continuous flow conditions (Figure 3b) versus the static sequential approach used in the cascade method (Figure 3a).

### 2.5. Flow Cytometry

To quantify the separation efficiency of the purification cascade, pre- and post-separation samples of the combined culture, plus non-mixed controls were diluted to 1:50 with BG-11 media and analyzed on a BD Accuri C6+ flow cytometer (BD Biosciences, Franklin Lakes, NJ, USA). After 30,000 measured events, gates were drawn around each specific cyanobacterial population based on the forward scatter and the autofluorescence peak (488 nm excitation laser and a 670LP detector (BD Biosciences, Franklin Lakes, NJ, USA)) of the axenic controls. The gates were used to determine cell counts (cells/μL) by the instruments BD Accuri C6 Plus Analysis software (BD Biosciences, NJ, Franklin Lakes, USA).

## 3. Results and Discussion

### 3.1. Simulation Results

**Figure 4:**
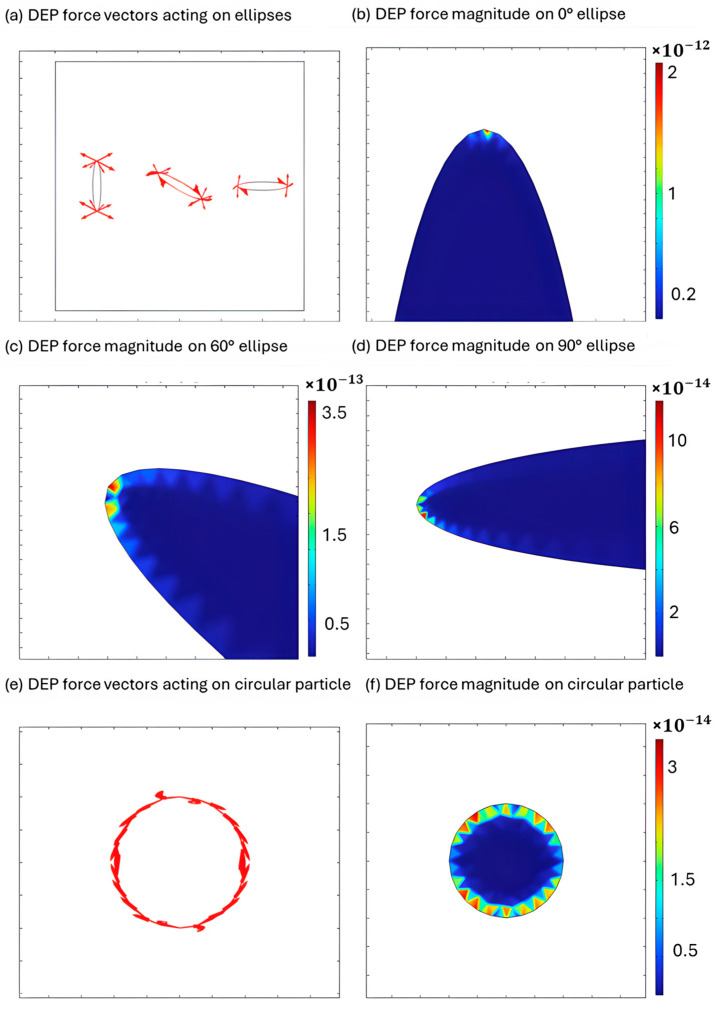
Simulated dielectrophoretic (DEP) behavior of elongated and spherical cyanobacterial cells. (**a**) DEP forces acting on elongated cells of various orientations; (**b**) DEP force for elliptical cells rotated at 0 degrees with respect to electrodes; (**c**) DEP force for elliptical cells rotated at 60 degrees with respect to electrodes; (**d**) DEP force for elliptical cells rotated at 90 degrees with respect to electrodes; (**e**) DEP force acting on a spherical cell; (**f**) magnitude of DEP force acting on a spherical cell.

To visualize the orientation-dependent DEP interactions, Figure 4a illustrates the simulated DEP force vectors acting on three elongated particles positioned side by side, each with a distinct orientation. The red arrows represent the magnitude and direction of the DEP force at discrete points around each particle. Notably, the vertically aligned particle (leftmost on Figure 4a) exhibits the strongest DEP response, as indicated by the large vector magnitudes distributed symmetrically around it. This symmetry suggests that the DEP forces are balanced, resulting in no net torque and a stable vertical orientation. In contrast, the diagonally and horizontally aligned particles experience weaker forces, generating a net torque that induces rotation toward vertical orientation. This simulated force distribution confirms the experimentally observed predominance of vertical orientation for the *Synechococcus*.

To quantify the orientation-dependent DEP response of elongated cells, the surface plots shown in Figure 4b–d represent the spatial distribution of the DEP force magnitude acting on elongated particles at different orientations 0°, 60°, and 90° with respect to the electrode, under a 10 MHz AC electric field. The force magnitude is calculated as (F_DEP_, _x_^2^ + F_DEP, y_^2^)^1/2^ and is visualized using a color scale. As seen in the plots, the vertical particle exhibits the highest DEP force magnitude, reaching the order of 10^−12^ N, as indicated by the red regions. The particle tilted at 60° shows a reduced maximum force to the order of 10^−13^ N, while the horizontally aligned particle experiences the weakest DEP response, peaking at the order of 10^−14^ N. The results confirm that vertical alignment of the *Synechococcus* is favored, and it produces the strongest DEP force in the vertical orientation of the cells.

To model the DEP behavior of a circular *Synechocystis* cell, Figure 4e,f demonstrate the simulated dielectrophoretic (DEP) force using Equations (1)–(3) under a 10 MHz, 20 Vpp electric field. The arrow plot shows that the DEP force vectors are directed radially outward from the particle center, indicating nDEP response where the particle is repelled from regions of high electric field intensity. Correspondingly, the surface plot of the DEP force magnitude reveals uniformly low force intensities around the particle boundary, with values peaking at the order of 10^−14^ N. This response contrasts with the higher DEP force observed for elongated particles. The weaker repulsive force of spherical cells like *Synechocystis* contrasted with stronger attractive force exhibited by *Synechococcus* suggests that DEP can be utilized for separation of the microbial mix. This result reinforces the basis for selective manipulation and separation based on morphological differences. These findings are consistent with the experimental observation presented below.

### 3.2. Evaluation of Feasibility of DEP-Based Cell Separation

**Figure 5:**
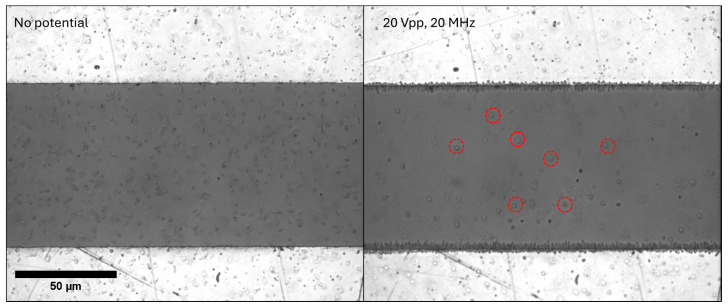
The separation of spherical *Synechocystis* (selected cells are circled in red) and elongated *Synechococcus* upon application of 20 Vpp and 20 MHz signal. The white areas are electrodes, and the dark area is the gap between electrodes.

The initial proof-of-concept experiments demonstrate the efficacy of electrokinetic separation of spherical *Synechocystis* and elongated *Synechococcus* cyanobacterial cells. When exposed to a non-uniform electric field, the elongated *Synechococcus* exhibited an attraction force towards the electrode edges, driven by the pDEP. This behavior is attributed to their morphology, as demonstrated by a COMSOL Multiphysics simulation discussed above. In contrast, the spherical *Synechocystis* were largely unaffected by the electric field, showing minimal response and remaining free to wash away during subsequent processes, as demonstrated in Figure 5. The experiments were conducted in BG-11 medium, the standard growth media for cyanobacteria. Low-ionic-strength DEP buffers are often used to tune conductivity, but such media can impose osmotic stress and may lead to membrane damage or lysis, particularly in cyanobacteria. They also introduce additional handling steps that can alter cellular properties. In this system, BG-11 already provides sufficient dielectric contrast between the two cyanobacterial strains to enable effective DEP separation. Therefore, operating directly in BG-11 preserves physiological conditions and avoids unnecessary cell stress. Before applying potential, the two types of cells are randomly distributed in the mixture, but upon the application of 20 Vpp 20 MHz signal the *Synechococcus* cells are attracted to the electrode edge due to pDEP, while the *Synechocystis* cells remain unaffected. The video recording of this experiment is provided in the Supplementary Data (Video S1).

The differences in electrokinetic behavior of two types of cells enabled their effective separation: while the elongated cells were drawn towards and concentrated at the electrode sites, the spherical cells, being less influenced by the applied electric field, were easily removed by the cross flow, confirming the feasibility of dielectrophoretic sorting based on morphological and dielectric properties of the cells. The exact AC frequency to control the separation varies depending on the mixture’s bio-condition. The separation of two cell types takes place between 1 and 20 MHz and 10 and 20 Vpp.

### 3.3. The Cascade Dielectrophoretic Separation

**Figure 6:**
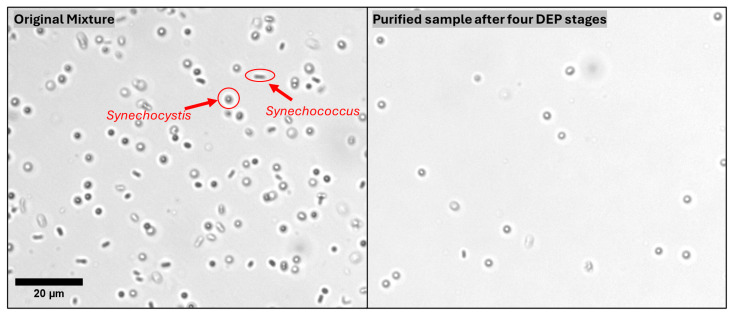
Images of cell populations before and after 4-stage cascade dielectrophoretic separation. The (**left**) panel shows the original mixture containing a high concentration of *Synechococcus* (elongated cells). The (**right**) panel displays the sample (at the same magnification) after four consecutive cascade IDEA stages, demonstrating a reduction in *Synechococcus*.

In order to enhance the separation of two types of cells the electrode cascade, connecting several stages of IDEAs was enacted where the cells separated out in one IDEA stage were loaded into the next microfluidic electrokinetic chip to enable further separation. The cascade configuration was selected because the current device geometry creates dead space above the electrode plane and allows accumulated *Synechococcus* to partially shield the electric field, both of which diminish separation efficiency within a single DEP cycle. Reintroducing the suspension to a fresh, unshielded electrode region restores the effective field gradient and enables additional refinement. The cascade separation system demonstrated the efficiency in purifying *Synechocystis* from an artificially mixed culture of *Synechocystis* and *Synechococcus*. Initially, the manual mixture contained a 2:1 ratio of *Synechocystis* to *Synechococcus* by volume. At each stage of the cascade, the electrode selectively captured *Synechococcus* under the DEP conditions while allowing *Synechocystis* to flow through. Figure 6 presents microscopic images of the sample before and after the cascade separation. The pre-cascade image Figure 6(left) illustrates a high concentration of *Synechococcus* within the sample, whereas Figure 6(right) demonstrates a significant degree of purification of the sample (reduction in *Synechococcus*) after four sequential stages of IDEAs DEP separation.

Two complementary separation strategies were tested: Sequential cascade resulted in higher degree of purification, but lower number of overall cells harvested (since some cells are lost in each separation stage), while QCF processing (equivalent to a single stage separation performed on many cells) results in a larger yield of useful cells but at lower degree of purification.

**Figure 7:**
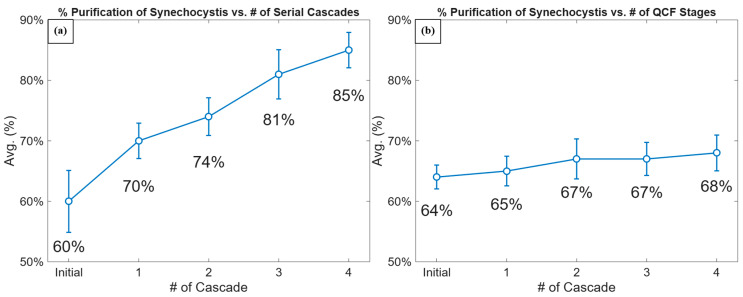
Purification efficiency of *Synechocystis* vs. number of IDEA stages sequential as a function of cascade number under two separation strategies: serial (**a**) and OCF (**b**). Each data point represents the average purification efficiency across replicates (n = 3), with error bars indicating the standard deviation.

To illustrate the purification trend across cascades, Figure 7a presents a plot of the cascade purification efficiency after each step, based on manual counting. This trend illustrates the progressive removal of *Synechococcus*, highlighting the effectiveness of each sequential cascade in improving overall purity of the cell suspension. The graph represents the impact of the cascade DEP purification process in achieving high levels of *Synechocystis* enrichment while eliminating *Synechococcus*. The degree of sample purification increases with the number of sequential stages.

Currently, a trade-off exists between achieving the highest possible purification of *Synechocystis* and retaining sufficient concentration for downstream bioproduction processes. While additional cascade enhances purity, it is also accompanied by the reduction in the total number of *Synechocystis*. Therefore, an optimal balance must be determined to maximize purity while preserving the target cell population.

In order to increase the number of captured cells, a QCF separation strategy was implemented. The QCF DEP separation involved processing cell suspension through DEP stages placed in QCF (equivalent to the multiple first cascade stages). The QCF separation outcome is reported in Figure 7b and Table 1. As expected, the QCF separation strategy resulted in a higher number of *Synechocystis* cells, albeit of lower purity. When high retention is a priority, such as in bioproduction workflows, the QCF method provides a practical alternative. Conversely, when maximum purity is required, such as in analytical or preparatory procedures, the serial method remains more effective. Some of the reported values show substantial variability across replicates, which reflects the limitations of the current adhesive-based channel structure. Some of the cells can get randomly trapped in the gaps between adhesive layers, leading to unintended loss of both target and non-target cells. However, the overall purification trend remains consistent across replicates.

### 3.4. Flow Cytometry Analysis

**Figure 8:**
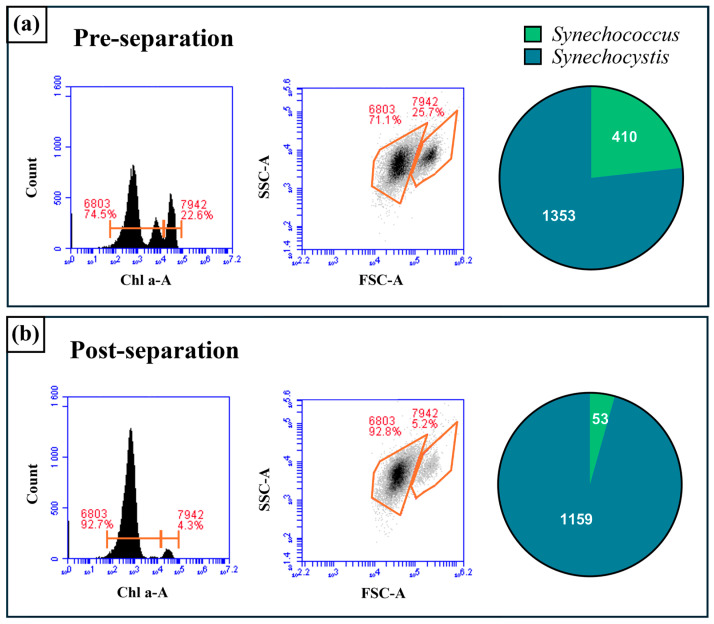
Illustration of the separation efficacy of the cascade purification process through flow cytometry analysis. The data are presented in three formats for both the pre-cascade (**a**) and post-cascade (**b**) samples: a histogram of fluorescence intensity (**left**), a forward scatter vs. side scatter dot plot (**middle**), and a pie chart representing the final cell population composition (**right**).

In the pre-cascade condition (Figure 8a), *Synechocystis and Synechococcus* are partially resolved, as shown in the fluorescence histograms and scatter plots derived from flow cytometry-based analysis. The cell population is comprised of 23.3% non-productive *Synechococcus* cells (410 counts) and 76.7% *Synechocystis* cells (1353 cells). Following the cascade purification (Figure 8b), the distribution shifts significantly as there is a decrease in the *Synechococcus* population and enrichment of the *Synechocystis* population. After separation, light scattering analysis of the samples via flow cytometry indicated that *Synechocystis* represented 95.6% (1159 counts) of the total population and *Synechococcus* was reduced to only 4.4% (53 counts) of the refined cell mix (i.e., more than five-fold reduction of less-productive cell strain fraction in the mixed culture).

This series of experiments demonstrates the high efficiency of the electrokinetic purification strategy in selectively isolating *Synechocystis*. Since *Synechocystis* is known to accumulate PHB [20,21,22], the selective enrichment of this strain in a mixed culture directly correlates with improved bioproduction performance. Notably, a single round of agar plating (Supplementary Data Figure S1) verified that cells remained viable after undergoing three cascades under various electric field conditions, further supporting the practicality of the method. However, for downstream applications focused on bioplastic recovery, cell viability is of less concern than achieving maximal enrichment. In summary, the cascade strategy substantially improves target cell purity, validating its utility in scalable biomanufacturing systems where strain-specific productivity is paramount.

The electrokinetic DEP cell sorting and separation technique presented here can be further enhanced for commercial applications by addressing several issues. The first issue relates to the loss of cells during the pipetting of cell suspension when transferring cells between stages of separation. Some of the cells can get trapped in the gaps between adhesive layers, leading to unintended loss of both target and non-target cells. As shown in Table 1, this results in a measurable decrease in the total *Synechocystis* population. Since there is no significant difference for the dead volume/sample loss between serial cascade method and QCF method (since the chips are identical), the comparison between two techniques is valid despite the sample loss and the trends legitimately indicate the preference of the serial cascade over QCF approach for cell capture/refinement. The number of stages used in the dielectrophoretic cascade were limited to four as the sample loss during the transfer between the IDEAs progressively affected the results and adding greater number of stages will leave smaller fraction of the original sample. The second limitation is the dead space within the channel of microfluidic IDEAs DEP, which reduces purification efficiency. Since DEP forces are the strongest near the interdigitated electrode surfaces, regions of the channel not exposed to strong electric fields, either laterally or vertically, fail to contribute meaningfully to separation. Cells passing through these areas experience low DEP forces, allowing non-target *Synechococcus* to escape sorting and lowering the overall selectivity.

It is envisioned that commercial microfluidic DEP chips will use injection molded chips that are ultrasonically bonded to minimize the dead volume and avoid the use of double-sided adhesive layers, addressing the limitations described above. For future integration of continuous-flow operation, controlled low-flow conditions can be introduced through mechanical pumping methods such as syringe pumps or through passive mechanisms including CD centrifugal microfluidic de-vice or capillary-driven flow [30,31]. These approaches are capable of maintaining creeping-flow regimes where hydrodynamic drag remains below the DEP trapping force, allowing effective separation without dislodging cells from the electrode region.

## 4. Conclusions

In this study, we demonstrated a novel cascade electrokinetic separation strategy for selectively enriching *Synechocystis* based on their dielectric properties using a DEP microfluidic platform. The results confirmed that integrating a multi-stage cascade effectively enhances the purity of high-PHB-producing cells, as evidenced by flow cytometry analysis. This enrichment strategy has promising implications for improving volumetric or mass productivity in microbial bioproduction, particularly for bioplastics such as PHB.

Furthermore, the viability of the sorted cells was maintained throughout the process, supporting the potential for downstream cultivation and bioproduction. Future efforts will focus on optimizing the electrode geometry to amplify the effective DEP trapping regions, as well as redesigning the microfluidic geometry to support required flow rates. These enhancements aim to increase the system’s throughput and transition from lab-scale proof-of-concept to industrial-scale mass-manufacturing applications.

## Figures and Tables

**Table 1:** The average percentage decreases of the *Synechocystis* population with use of four DEP stages in serial cascade and in QCF DEP separation. The serial DEP cascade results in a larger number of lost *Synechocystis* cells (especially given the compounded loss after each stage of the refinement process). Values represent mean ± standard deviation (%) (n = 3).

Stage #	Serial Cascades (%)	QCF Stages (%)
Stage #1	39.2 ± 35.2	8.3 ± 9.7
Stage #2	12.2 ± 7.5	29.3 ± 20.4
Stage #3	23.8 ± 14.8	11.0 ± 11.8
Stage #4	24.6 ± 19.1	28.4 ± 16.8

## Data Availability

Data will be made available on request.

## Supplementary Materials

The following supporting information can be downloaded at: https://www.mdpi.com/article/10.3390/mi16121402/s1, Figure S1: Agar plates show cyanobacterial growth after electrokinetic treatment; Video S1: 50× Demonstration of rod and round mixture separation in non glucose BG-11, separation at around 5–7 MHz, 20 Vpp (https://drive.google.com/file/d/1XKP9svDg-_1A5rwOvNWes-bvMre9rDE3/view, accessed on 12 December 2025).
