# Supplementary material for: Cascade Dielectrophoretic Separation for Selective Enrichment of Polyhydroxybutyrate (PHB)-Producing Cyanobacterium Synechocystis sp. PCC 6803
Source: Micromachines (Basel). 2025 Dec 12;16(12):1402. doi: 10.3390/mi16121402 (PMC12734510; doi:10.3390/mi16121402)
Supplement: Supplementary file 1 [file micromachines-16-01402-s001.zip › micromachines-3977193-supplementary.pdf]

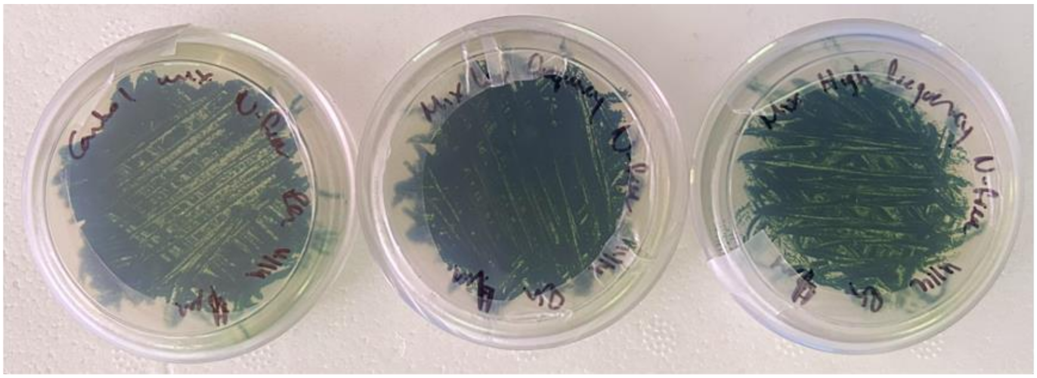

Figure S1. Agar plates show cyanobacterial growth after electrokinetic treatment. The control (left) is the untreated mixture of *Synechocystis* and *Synechococcus*. The middle and right samples were processed by low (4 Vpp, 5 min) and high (20 Vpp, 3 min) electric fields, respectively. Proliferation across all groups indicates that electrokinetic separation preserves cell viability.
